# Low risk of nosocomial severe acute respiratory syndrome-coronavirus-2 infection in patients with liver disease admitted to a hepatology unit at an academic hospital: A single-center experience

**DOI:** 10.1007/s12664-022-01241-8

**Published:** 2022-06-30

**Authors:** Pierluigi Toniutto, Federica D’Aurizio, Sara Cmet, Annarosa Cussigh, Edmondo Falleti, Carlo Fabris, Emma Sartor, Ezio Fornasiere, Elisa Fumolo, Davide Bitetto, Francesco Curcio

**Affiliations:** 1 Hepatology and Liver Transplantation Unit, Azienda Sanitaria Universitaria Integrata, University Hospital, Udine, Italy; 2 Clinical Pathology, Azienda Sanitaria Universitaria Integrata, University Hospital, Udine, Italy; 3 Microbiology, Azienda Sanitaria Universitaria Integrata, University Hospital, Udine, Italy

**Keywords:** Hepatocellular carcinoma, Immunoglobulin G, Immunoglobulin M, Immunosuppression, Liver cirrhosis, Liver transplantation, Nasopharyngeal swab, Nosocomial infection, Reverse transcriptase polymerase chain reaction, SARS-CoV-2 infection, Vaccination

## Abstract

**Background:**

Patients with liver disease may be at increased risk of severe acute respiratory syndrome-coronavirus-2 (SARS-CoV-2) infection due to immune dysfunction. However, the risk of nosocomial SARS-CoV-2 infection in these patients remains unknown. This study aimed to determine whether patients with liver disease are at an increased risk of nosocomial transmission of SARS-CoV-2 infection upon admission to the hospital for diagnostic or therapeutic procedures.

**Methods:**

The study prospectively enrolled 143 patients who were admitted at least once to the hepatology unit at our hospital; 95 patients (66%) were admitted at least twice during the study period. History of past symptomatic SARS-CoV-2 exposure was assessed on the day before hospital admission via an interview. Patients were evaluated for active SARS-CoV-2 infection via real-time reverse transcription–polymerase chain reaction (RT-PCR) performed on nasopharyngeal swabs and tests for serum anti-SARS-CoV-2 immunoglobulin M (IgM) and immunoglobulin G (IgG) antibodies.

**Results:**

None of the patients enrolled tested positive for SARS-CoV-2 infection by RT-PCR at the first or the second clinical evaluation. One patient who had previously received a liver transplant and who had a history of symptomatic SARS-CoV-2 infection that occurred 4 months before hospital admission tested positive for anti-SARS-CoV-2 IgG but not IgM antibodies at each of the two hospital admissions.

**Conclusions:**

The results of our study suggest that patients with liver disease are at no increased risk of nosocomial SARS-CoV-2 infection. These data support the policy of maintaining clinical hospital checks that will be necessary until or possibly even after the completion of the current SARS-CoV-2 vaccination campaign.

Bullet points of study highlights ***What is already known?***

- The clinical severity of severe acute respiratory syndrome-coronavirus-2 (SARS-CoV-2) infection in patients with liver diseases is higher compared to general population.

***What is new in this study?***

- Patients with liver disease seem to be at no increased risk of nosocomial SARS-CoV-2 infection.

***What are the future clinical and research implications of the study findings?***

- The maintenance of clinical hospital checks that will be necessary until or possibly even after the completion of the current SARS-CoV-2 vaccination campaign is advisable.

## Introduction

The new coronavirus pathogen, severe acute respiratory syndrome-coronavirus-2 (SARS-CoV-2), has been identified as the cause of corona virus disease-19 (COVID-19). One hallmark of SARS-CoV-2 is its highly contagious nature. Recent studies have revealed that SARS-CoV-2 is easily spread by respiratory droplets transmitted by close contact with infected individuals that may persist on surfaces for up to 3 days [1]. The infection may be asymptomatic or associated with a few influenza-like symptoms, including fever, nasal congestion, and cough. Anosmia and ageusia have also been reported in a large fraction of SARS-CoV-2-infected patients. However, some infected patients may deteriorate rapidly and can develop bilateral interstitial pneumonia. This condition is often associated with severe respiratory distress, multi-organ failure, and death [2]. Several demographic and clinical factors have been associated with these more severe outcomes of SARS-CoV-2, including older age, frailty indicators, and co-morbidities including overweight, diabetes, cardiac, and respiratory chronic diseases [3]. Patients with liver disease including those who have received liver transplants or those with advanced-stage cirrhosis often experience more aggressive SARS-CoV-2 infection and have an increased mortality rate compared to the general population [4].

Despite robust infection-control efforts, hospital-acquired (nosocomial) SARS-CoV-2 infections have been reported in healthcare workers and patients with various diseases [5–7]. Patients with advanced liver disease may be at increased risk of infection due to cirrhosis-induced immune dysfunction [8]. This may also be the case for patients with autoimmune liver diseases and/or those who have received liver transplants who are maintained on immunosuppressive therapies [9]. For these reasons, patients diagnosed with liver disease (as well as individuals in the general public) have expressed increasing reluctance to be admitted to a hospital for diagnostic tests and/or critical treatments. This may account for the significant reduction in acute hospital admissions and may also have contributed to the recently observed higher than average mortality rates for patients with underlying liver disease [10]. Furthermore, the management and surveillance of patients with advanced liver disease, as well as ongoing care for those who have undergone liver transplantation, are typically carried out in designated hepatology units located in centralized hospitals. These hospitals are frequently in areas that are COVID-19 “hotspots.” This may place hospital inpatients with liver diseases at increased risk of acquiring nosocomial infection [11].

However, the real risk of acquiring a nosocomial SARS-CoV-2 infection in this patient cohort remains unknown. The present study aimed to determine whether patients with liver disease or with a history of liver transplantation may be at an increased risk of nosocomial transmission of SARS-CoV-2 infection upon admission to a hospital for diagnostic or therapeutic procedures.

## Methods

### Patients

We enrolled all consecutive patients carrying a diagnosis of liver disease who were admitted to the hepatology and liver transplantation unit of the University of Udine’s academic hospital in Italy, at least once from March 1 to October 31, 2020. All patients expressed their consent to participate in the study, the protocol of which was reviewed and approved by the hospital review board, following the Declaration of Helsinki. At enrollment, patient demographics, as well as clinical and laboratory data, were prospectively recorded, and each patient’s medication list was carefully reviewed. All authors had access to the study data and reviewed and approved the final manuscript.

### Assessment of SARS-CoV-2 infection

All patients participated in detailed phone interviews on the day before hospital admission to assess the presence of respiratory symptoms and any potential contact with persons infected with SARS-CoV-2 during the previous 15 days.

In all patients active SARS-CoV-2 infection was assessed by the collection of nasopharyngeal swabs followed by real-time reverse transcription–polymerase chain reaction (RT-PCR) testing (LightMix® SarbecoV E-gene plus EAV control using real-time Roche LightCycler®480, TIB Molbiol, Roche, Swiss), as recommended by clinical guidelines [12, 13], and by measurement of serum immunoglobulin M (IgM) and immunoglobulin G (IgG) antibodies directed against the SARS-CoV-2 receptor binding Spike protein S1 (ADVIA Centaur® SARS-CoV-2 Total chemiluminescent immunoassay run on a Centaur XP automatic analyzer, Siemens Healthcare, Malvern (Pennsylvania), USA). Nasopharyngeal swabs were placed immediately in viral transport medium and sent under refrigerated conditions to the clinical microbiology laboratory. The results of RT-PCR testing were available within 12 h so that all patients with negative test results could be admitted to the hepatology unit on the following day.

To determine whether a patient had developed serum antibodies against SARS-CoV-2, blood samples were collected and separated. The serum fraction was stored at −80 °C until the day of testing. Each positive sample was re-tested both with and without pretreatment with heterophilic blocking tubes (HBTs) and with non-specific antibody blocking tubes (NABTs, Scantibodies Laboratories, Villebon sur Yvette, France) to eliminate interference from heterophilic or nonspecific antibodies. Positive samples were retested with iFlash-SARS-CoV-2 IgG and iFlash-SARS-CoV-2 IgM chemiluminescent immunoassay on an iFlash1800 Immunoassay Analyzer (Shenzhen Yhlo Biotech, Shenzhen, China).

To prevent nosocomial SARS-CoV-2 infection, several in-hospital control measures were adopted. Body temperature for each patient was recorded before the entrance in the clinic. All admitted patients were provided with a Filtering Facepiece type 2 (FFP2) mask, latex gloves, and a disposable synthetic material gown to wear over the clothing. Patients had a single station (chair or bed) available, depending on the type of procedure performed (blood sampling or invasive procedures), and had no physical contact with other patients. The medical and nursing staff wore a synthetic gown, mask, visor, and latex gloves. Gloves and gown were changed for each patient visited.

### Statistical analysis

Statistical analysis was performed using the Bio-Medical Data Package (BMDP) statistical software program (Statistical Solutions -Ltd, Cork, Ireland). Continuous variables are presented as medians with interquartile range (IQR) and were analyzed using the Mann-Whitney *U* test. Categorical variables are presented as frequencies (%) and were analyzed using the Chi-squared test.

## Results

### Patients

The study enrolled 143 consecutive patients with liver disease (70% males, mean age of 62 years) who were admitted either as outpatients or inpatients to the hepatology unit of our hospital. Among the 143 patients who were admitted for a first clinical evaluation, 95 patients also underwent a follow-up (second) evaluation. The median time from the first to the second evaluation was 95 days (IQR, 40–140 days). Outpatient admissions were typically for radiologic and/or laboratory examinations and drug infusion. Inpatient admissions were primarily for invasive diagnostic or therapeutic procedures (e.g. liver biopsy or angiography) or to perform loco-regional treatment for hepatocellular carcinoma (HCC). The main demographic and clinical characteristics of the patients who were admitted for a first clinical evaluation are presented in Table 1. Among the 143 patients enrolled, 38 (26.6%) had received a liver transplant. The main etiologies of liver disease in this patient cohort were alcohol-related conditions and HCC. We identified no significant differences regarding the etiologies of liver diseases between patients who had undergone transplantation procedures and those who had not. Among the patients who have not received liver transplants, 76/105 (72.4%) presented with cirrhosis and 13/105 (12.4%) were taking immunosuppressive drugs to treat autoimmune hepatitis. Patients who had undergone liver transplants were admitted as outpatients more frequently than were those who had not received transplants, typically for radiologic and/or laboratory examinations (34 of 38 [89.5%] vs. 76 of 105 [72.4%], *p* = 0.032). We identified no significant differences between these two groups with respect to reports of SARS-CoV-2, symptoms before admission and/or an earlier risk of exposure, which were recorded in 4/38 (10.5%) of the patients who had undergone liver transplants and 8/105 (7.6%) of those who had not (*p* = 0.580).

**Table 1:** Primary demographic and clinical characteristics of patients admitted to the hepatology unit for the first time between March 1 and October 31, 2020 (*n* = 143)

	Liver transplanted (*n* = 38)	Non-transplanted (*n* = 105)	*p*
Age, years	61.7 (56.2–65.8)	61.7 (54.1–67.5)	0.891
Male gender, *n* (%)	27 (71.0)	71 (67.6)	0.696
Time from LT, months	10 (6 – 24)	-	-
Etiology of liver disease*			
Viral	6 (15.8%)	11 (10.5)	0.386
Alcoholic	10 (26.3%)	31 (29.5%)	0.708
HCC	14 (36.8%)	23 (21.9%)	0.072
Other	8 (21.1%)	40 (38.1%)	0.057
Cirrhosis	-	76 (72.4%)	-
MELD-Na score	-	11 (8–15)	-
Child-Pugh score	-	6 (5–7)	-
Immunosuppressive therapy	38 (100%)	13 (12.4%)	-
Use of ACE inhibitors	2 (5.3%)	16 (15.2%)	0.112
Type of hospital admission			
Outpatient	35 (92.1%)	93 (88.6%)	0.542
Inpatient	3 (7.9%)	12 (11.4%)	
Reasons for hospital admission			
Radiology, laboratory	34 (89.5%)	76 (72.4%)	0.032
Drug infusion	0 (0.0%)	9 (8.6%)	0.062
Invasive procedure	4 (10.5%)	20 (19.0%)	0.228
Corona virus disease-19 symptoms or contact risk**	4 (10.5%)	8 (7.6%)	0.580

Continuous variables are presented as median (followed by the interquartile range in brackets) and categorical variables as frequencies (%) for patients who had received liver transplants (liver transplanted) and patients who had not received liver transplants (non-transplanted). Continuous variables were evaluated with the Mann-Whitney *U* test and categorical variables were evaluated using the Pearson Chi-square test to compare liver transplanted and non-transplanted patients

*LT* liver transplantation, *MELD* model for end-stage liver disease, *HCC* hepatocellular carcinoma, *ACE* angiotensin-converting enzyme, *in LT patients the etiology of liver disease refers to that recorded at the time that the transplant was requested; **symptoms including fever, chills, cough, or contact with persons infected with severe acute respiratory syndrome-coronavirus-2 during the previous 15 days

The primary laboratory parameters that were recorded at the first clinical evaluation of all patients are shown in Table 2. As anticipated, patients with liver transplants presented with significantly higher serum creatinine levels compared to those who had not received transplants. In contrast, patients who have not received liver transplants presented with significantly higher levels of serum bilirubin and international normalized ratios (INRs) and significantly lower platelet counts and serum sodium levels compared to transplanted patients.

**Table 2:** Primary hematological and biochemical laboratory parameters of patients admitted to the hepatology unit for the first time between March 1 and October 31, 2020

	Liver transplanted (*n* = 38)	Non-transplanted (*n* = 105)	*p*
Hemoglobin (g/dL)	13.0 (11.3–14.5)	12.8 (11.6–14.1)	0.978
Platelets (n*1000/μL)	154 (133–203)	110 (72–191)	0.016
Leukocytes (n*1000/μL)	4.78 (4.18–5.88)	4.30 (3.22–5.87)	0.206
Lymphocytes (n*1000/μL)	1.23 (0.75–1.74)	1.09 (0.70–1.60)	0.420
INR	1.07 (1.03–1.14)	1.18 (1.05–1.34)	0.002
Sodium (mmol/L)	140 (139–142)	139 (137–141)	<0.001
Creatinine (mg/dL)	1.12 (0.93–1.35)	0.91 (0.74–1.03)	<0.001
Total bilirubin (mg/dL)	0.60 (0.41–1.10)	1.06 (0.61–2.10)	<0.001

Continuous variables are presented as median (followed by the interquartile range in brackets) for patients who had received liver transplants (liver transplanted) and patients who had not received liver transplants (non-transplanted). Continuous variables were evaluated using the Mann-Whitney *U* test

*INR* international normalized ratio

Ninety-five patients, of whom 31 (32.6%) had received liver transplants, were admitted for a second clinical evaluation. Table 3 summarizes the main demographic and clinical characteristics of these patients. The only significant difference between the two groups was that a considerably longer period of time elapsed between the first and the second clinical evaluation among those who received liver transplants compared to those who did not (118 vs. 72 days, *p* < 0.02). As observed in patients evaluated at their first visit, we found no significant differences between those who had received liver transplants (2/31; 6.5%) and those who had not (6/64; 9.4%) when we examined these patient cohorts for symptoms including fever, chills, or cough or contacts with persons infected with SARS-CoV-2 during the 15 days immediately preceding the second clinical evaluation (*p* = 0.630).

**Table 3:** Primary demographic and clinical characteristics of patients admitted to the hepatology unit for the second time between March 1 to October 31, 2020 (*n* = 95)

	Liver transplanted (*n* = 31)	Non-transplanted (*n* = 64)	*p*
Days from the first clinical evaluation	118 (82–140)	72 (40–128)	0.016
Age, years	60.6 (52.9–65.7)	62.0 (55.8–67.2)	0.427
Male gender *n* (%)	21 (67.7)	46 (71.9)	0.679
Time from LT, months	12 (8–20)	-	-
Etiology of liver disease*			
Viral	4 (12.9%)	7 (10.9%)	0.779
Alcoholic	9 (29.0%)	25 (39.1%)	0.339
HCC	11 (35.5%)	12 (18.7%)	0.074
Other	7 (22.6%)	20 (31.3%)	0.380
Cirrhosis	-	48 (75.0%)	-
MELD-Na score	-	11 (9–16)	-
Child-Pugh score	-	7 (5–8)	-
Immunosuppressive therapy	31 (100%)	7 (10.9%)	-
Use of ACE inhibitors	1 (3.2%)	7 (10.9%)	0.204
Type of hospital admission			
Outpatients	29 (93.5%)	61 (95.3%)	0.718
Inpatients	2 (6.5%)	3 (4.7%)	
Reasons for hospital admission			
Radiology, laboratory	28 (90.3%)	51 (79.7%)	0.194
Drug infusions	0 (0.0%)	5 (7.8%)	0.110
Invasive procedures	3 (9.7%)	8 (12.5%)	0.687
Corona virus disease-19 symptoms or contact risk**	2 (6.4%)	6 (9.4%)	0.630

Continuous variables are presented as median (followed by the interquartile range in brackets) and categorical variables as frequencies (%). Mann-Whitney *U* test was used to evaluate continuous variables and the Pearson Chi-square test was used to evaluate categorical variables for patients who received liver transplants (liver transplanted) and those who have not (non-transplanted)

*LT* liver transplantation, *MELD* model for end-stage liver disease, *HCC* hepatocellular carcinoma, *ACE* angiotensin-converting enzyme. **In LT patients, the etiology of liver disease refers to the diagnosis recorded at the time of transplant; **symptoms including fever, chills, or cough or contacts with persons infected with severe acute respiratory syndrome-coronavirus-2 during the previous 15 days

The primary laboratory parameters obtained from these patients at the second clinical evaluation are shown in Table 4. Similar to those recorded in the first clinical evaluation, patients who had received a liver transplant presented higher serum creatinine levels compared to those who had not received transplants, while the latter group presented higher serum bilirubin and INR levels and lower platelet counts and serum sodium levels compared to those in the former group.

**Table 4:** Primary hematological and biochemical parameters of patients admitted to the hepatology unit for a second time between March 1 and October 31, 2020 (*n* = 95)

	Liver transplanted (*n* = 31)	Non-transplanted (*n* = 64)	*p*
Hemoglobin (g/dL)	12.8 (11.7–13.8)	12.3 (11.4–13.8)	0.321
Platelets (n*1000/μL)	149 (120–182)	102 (61–181)	0.012
Leukocytes (n*1000/μL)	4.53 (3.55–5.91)	4.43 (3.16–5.26)	0.472
Lymphocytes (n*1000/μL)	1.17 (0.65–1.61)	0.90 (0.59–1.40)	0.296
INR	1.05 (1.02–1.13)	1.21 (1.06–1.32)	0.001
Sodium (mmol/L)	141 (139–142)	139 (137–141)	0.033
Creatinine (mg/dL)	1.19 (0.94–1.31)	0.93 (0.76–1.13)	0.013
Total bilirubin (mg/dL)	0.56 (0.49–0.80)	1.08 (0.66–1.77)	<0.001

Continuous variables are presented as median (followed by the IQR in brackets) for patients who had received liver transplants (liver transplanted) and patients who had not received liver transplants (non-transplanted). Continuous variables were evaluated using the Mann-Whitney *U* test.

*INR* international normalized ratio

### Incidence of nosocomial SARS-CoV-2 infection

None of the patients admitted for a first or a second clinical evaluation had positive nasopharyngeal swab RT-PCR tests for SARS-CoV-2. One outpatient had a positive anti-SARS-CoV-2 IgG but not IgM before the first clinical evaluation. This patient was a 67-year-old male who had received a liver transplant for decompensated hepatitis B-related cirrhosis 8 years before this hospital admission. At his interview, he reported a history of symptomatic SARS-CoV-2 infection 4 months before this admission. Serum anti-SARS-CoV-2 IgG antibodies were detected again after the 2 months that had elapsed between his first and second clinical evaluation, although the results of the RT-PCR test for SARS-CoV-2 in nasopharyngeal swabs remained negative throughout. No anti-SARS-CoV-2 antibodies were detected in any of the remaining 11 patients who were at increased risk for SARS-CoV-2 infection during the 15 days before a first admission (3 who had undergone liver transplantation and 8 who had not) or any of the 7 remaining patients (1 who had undergone liver transplantation and 6 who had not) who were at increased risk during the 15 days before the second admission.

## Discussion

None of the patients enrolled in our study presented with or developed clinical or laboratory-diagnosed SARS-CoV-2 infection. This result is somewhat remarkable given the prevalence of SARS-CoV-2 in Italy during this period. In the periodical report of the Italian National Institute of Health (www.epicentro.iss.it), (from February 1st to October 30th, 2020, a total of 12,043 cases of SARS-CoV-2 infection were documented in the general population of the Italian region where the study was conducted, leading to an incidence of 1%. In a recent report from Japan, the prevalence of nosocomial SARS-CoV-2 infection in patients with liver diseases evaluated from March to May 2020 was 0.17% [14]. While the prevalence of SARS-CoV-2 infection appears to be higher in this otherwise analogous patient cohort, this earlier study used two different immunoassay tests to detect anti-SARS-CoV-2 serum antibodies. We note that only one of their 300 patients tested positive for SARS-CoV-2 using the electrochemiluminescence immunoassay, which is similar to the test used in our study, while two patients tested positive using the immunochromatographic test. Of critical importance, none of these three patients tested positive for SARS-CoV-2 on both tests simultaneously. This may be due to the different sensitivities and specificities of the assays. All the patients enrolled in our study tested negative using the nasopharyngeal swab RT-PCR assay, which virtually excludes the possibility that negative antibody tests could be attributed to early-stage infection. Furthermore, the median time of approximately 3 months between the first and second clinic admissions reduces the likelihood that an anti-SARS-CoV-2 antibody titer that developed in the interim would have already disappeared even in patients who may have experienced an asymptomatic SARS-CoV-2 infection. Although data are still conflicting, it seems that the immune response after SARS-CoV-2 infection is similar, albeit probably slightly delayed, in patients with advanced liver disease or in transplant recipients compared to immunocompetent subjects [15]. Furthermore, the seroprevalence of SARS-CoV-2 infection in liver transplanted patients is similar to that recorded in the general population [16], suggesting that the specific immunosuppressive agents used to maintain patients who have undergone liver transplantation do not result in diminished antibody production.

The clinical outcome of COVID-19 in liver transplant recipients and in patients with cirrhosis seems to be not the same. In liver transplant recipients, the mortality rate due to SARS-CoV-2 infection was 18%, which was lower than in the matched general population [17]. However, in a recent study conducted in Italy in patients with advanced liver disease infected by SARS-CoV-2, the hospitalization rate was 96% and the 30-day mortality rate was 34%. Independent predictors of mortality were the severity of liver diseases calculated by model for end-stage of liver disease (MELD) score, and the development of extrahepatic organ failures [18]. These results were confirmed in a larger European multicenter study, which enrolled candidates for liver transplantation [19]. These observations suggest that vulnerable patient cohorts like patients with advanced liver diseases and liver transplant recipients should be protected from individuals with potential SARS-CoV-2 exposure or infection. Thus, in these patients, the benefits of maintaining patient care must be weighed against the risk of infection. Our data suggest that there is no increased risk of nosocomial SARS-CoV-2 infection in patients with liver disease or those who have received liver transplant compared to individuals in the general population. These results suggest the necessity of maintaining clinical hospital protocols that serve to present SARS-CoV-2 transmission until or possibly even after the completion of the current SARS-CoV-2 vaccination campaign.

The results of our study are limited by the single-center study design and by the small number of patients enrolled. Thus, our findings cannot be generalized. However, all patients were evaluated prospectively, and all were evaluated using the same testing protocol, which are both factors that add strength to our findings. The absence of a control group, e.g. patients with extrahepatic diseases who were also screened for the risk of nosocomial SARS-CoV-2 infection at the same time and in the same location as the patients enrolled in our study represents a further limitation. However, given the very low percentage positivity for SARS-CoV-2 infection identified among those in our immunosuppressed patient cohorts, it is unlikely that the risk of nosocomial SARS-CoV-2 infection would be significantly different among those who are otherwise immunocompetent. The adoption of qualitative instead of quantitative assessment of antibody response may be criticized. Since the sensitivity of the qualitative antibody test is comparable to the quantitative one [20], we believe it is unlikely that there was an underestimation of the incidence of seropositivity to SARS-CoV-2 infection in our study population.

## Declarations

### Disclaimer

The authors are solely responsible for the data and the contents of the paper. In no way, the Honorary Editor-in-Chief, Editorial Board Members, the Indian Society of Gastroenterology or the printer/publishers are responsible for the results/findings and content of this article.
